# Circulating tumor DNA dynamics and response to immunotherapy in colorectal cancer

**DOI:** 10.3892/mco.2022.2533

**Published:** 2022-03-30

**Authors:** Jun Gong, Francesca Aguirre, Dennis Hazelett, Rocio Alvarez, Lisa Zhou, Andrew Hendifar, Arsen Osipov, Karen Zaghiyan, May Cho, Alexandra Gangi, Megan Hitchins

**Affiliations:** 1 Department of Medicine, Division of Hematology and Oncology, Samuel Oschin Comprehensive Cancer Institute, Cedars-Sinai Medical Center, Los Angeles, CA 90048, USA; 2 Department of Biomedical Sciences, Cedars-Sinai Medical Center, Los Angeles, CA 90048, USA; 3 Department of Surgery, Samuel Oschin Comprehensive Cancer Institute, Cedars-Sinai Medical Center, Los Angeles, CA 90048, USA; 4 Division of Hematology and Oncology, Department of Medicine, University of California, Irvine, CA 92697, USA

**Keywords:** colorectal cancer, circulating tumor DNA, immunotherapy, microsatellite stable, microsatellite instability high

## Abstract

Circulating tumor DNA (ctDNA) is increasingly being investigated as a tool to detect minimal residual disease in resected, stage I-III colorectal cancer. Recent ctDNA studies have indicated that detection of ctDNA following surgery for resectable colorectal cancer confers a significantly higher risk of recurrence than those with negative ctDNA postoperatively. In those with postoperative ctDNA positivity, clearance of minimal residual disease with adjuvant chemotherapy is a positive prognostic indicator. Lastly, ctDNA has demonstrated superior sensitivity to the conventional blood tumor marker carcinoembryonic antigen (CEA) and can offer median lead times of up to 11 months for radiographic detection of recurrence during the surveillance of resected, stage I-III colorectal cancer. In metastatic colorectal cancer (mCRC), there is growing evidence to suggest that plasma ctDNA can be used to monitor tumor response to conventional chemotherapy as well. The present case series demonstrated that plasma ctDNA is a predictor of tumor response to immunotherapy in patients with mCRC that are microsatellite stable or microsatellite instability high. Plasma ctDNA could serve as a dynamic marker of immunotherapy response even in colorectal tumors that were CEA non-producers. Overall, these findings add to ongoing efforts to establish the role of plasma ctDNA in monitoring response to immunotherapy in CRC.

## Introduction

Immunotherapy in the form of immune checkpoint inhibitors have become U.S. Food and Drug Administration (FDA) approved in the treatment of metastatic colorectal cancer (mCRC) that is microsatellite instability-high (MSI-H) or mismatch repair deficient (dMMR) in both the first-line (1) and treatment-refractory settings (2-5). Despite promising overall response rates (ORRs) and durable responses demonstrated with immunotherapy in mCRC, carcinoembryonic antigen (CEA) remains the only conventional blood-based tumor marker to assess systemic therapy responses. A more widely applicable blood-based measure of tumor response to systemic therapies inclusive of immunotherapy could prove useful given that up to 34% of patients with CRC are CEA non-producers (6). Recently, circulating tumor DNA (ctDNA) has been recognized as a reliable tool in oncology that appears more sensitive to changes in tumor burden and monitoring of tumor response to systemic therapies than conventional approaches in CRC (7). In this case series, we report the utility of serial plasma ctDNA analyses using a modified Epi proColon^®^ 2.0 CE (Epigenomics AG) assay for ctDNA testing on the methylated *SEPTIN9* gene (mSEPT9) as a dynamic marker of response to immunotherapy in mCRC (8). The Epi proColon assay was modified for cell free DNA extraction from just 1 ml plasma with semi-quantification of mSEPT9 ctDNA levels that were calculated as previously described (8). Serial blood collections for mSEPT9 testing were performed under an IRB-approved protocol Pro00054104.

## Case report

### Case 1

**Figure 1:**
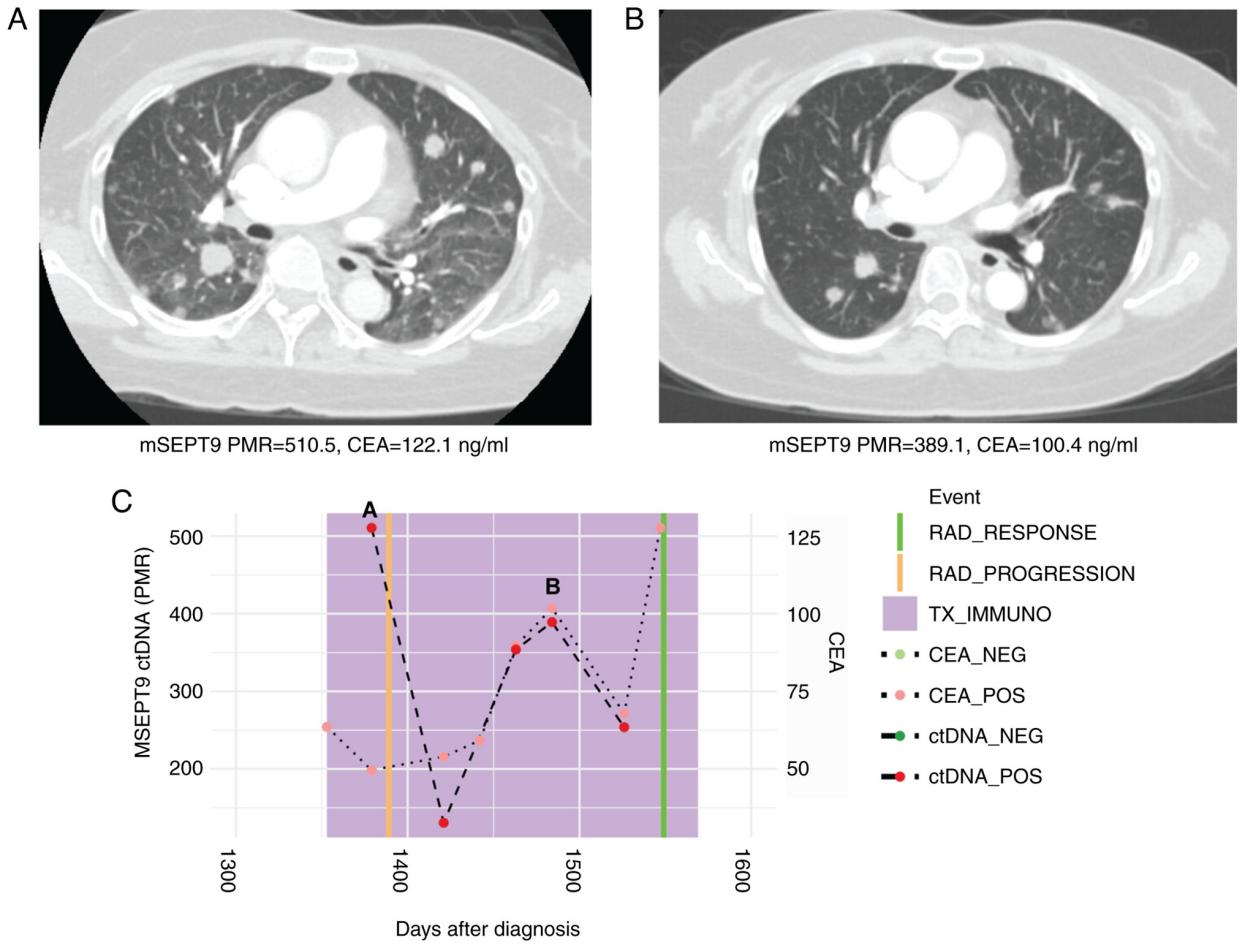
Case 1 with microsatellite stable metastatic CRC shows decline in ctDNA levels with response to immunotherapy. (A) CT scan at initiation of immunotherapy treatment showed widespread pulmonary metastases. Beneath, mSEPT9 ctDNA and CEA levels from blood drawn at this timepoint. (B) CT scan after four cycles of third-line regorafenib and pembrolizumab showed marked reduction in size of pulmonary metastases. Beneath, ctDNA and CEA levels from blood drawn at this timepoint were decreased. (C) Timeline of mSEPT9 and CEA levels over immunotherapy course. The timepoints matching CT panels A and B above are shown on the timeline graph. Reduced mSEPT9 ctDNA and CEA levels between initiation, and post-cycle four of immunotherapy correlated with response to treatment. mSEPT9, methylated *SEPTIN9* gene; PMR, percentage of methylation reference; CEA, carcinoembryonic antigen; RAD, radiologic; TX_IMMUNO, treatment with immunotherapy; NEG, negative; POS, positive; ctDNA, circulating tumor DNA; CT, computed tomography.

A 60-year-old woman with treatment-refractory microsatellite stable (MSS) mCRC to the liver, lungs, and pelvis was treated with third-line regorafenib (80 mg oral once a day for 21 days every 28-day cycles) and pembrolizumab (200 mg intravenously every 3 weeks). Plasma ctDNA analysis of mSEPT9 at treatment initiation showed positivity with a percentage of methylation reference (PMR) of 510.50. After 4 cycles of pembrolizumab and regorafenib, the mSEPT9 PMR value decreased to 389.12 correlating to a radiographic response on computed tomography (CT) scan (Fig. 1). Carcinoembryonic antigen (CEA) levels similarly decreased from 122.1 to 100.4 ng/ml along these same timepoints.

### Case 2

**Figure 2:**
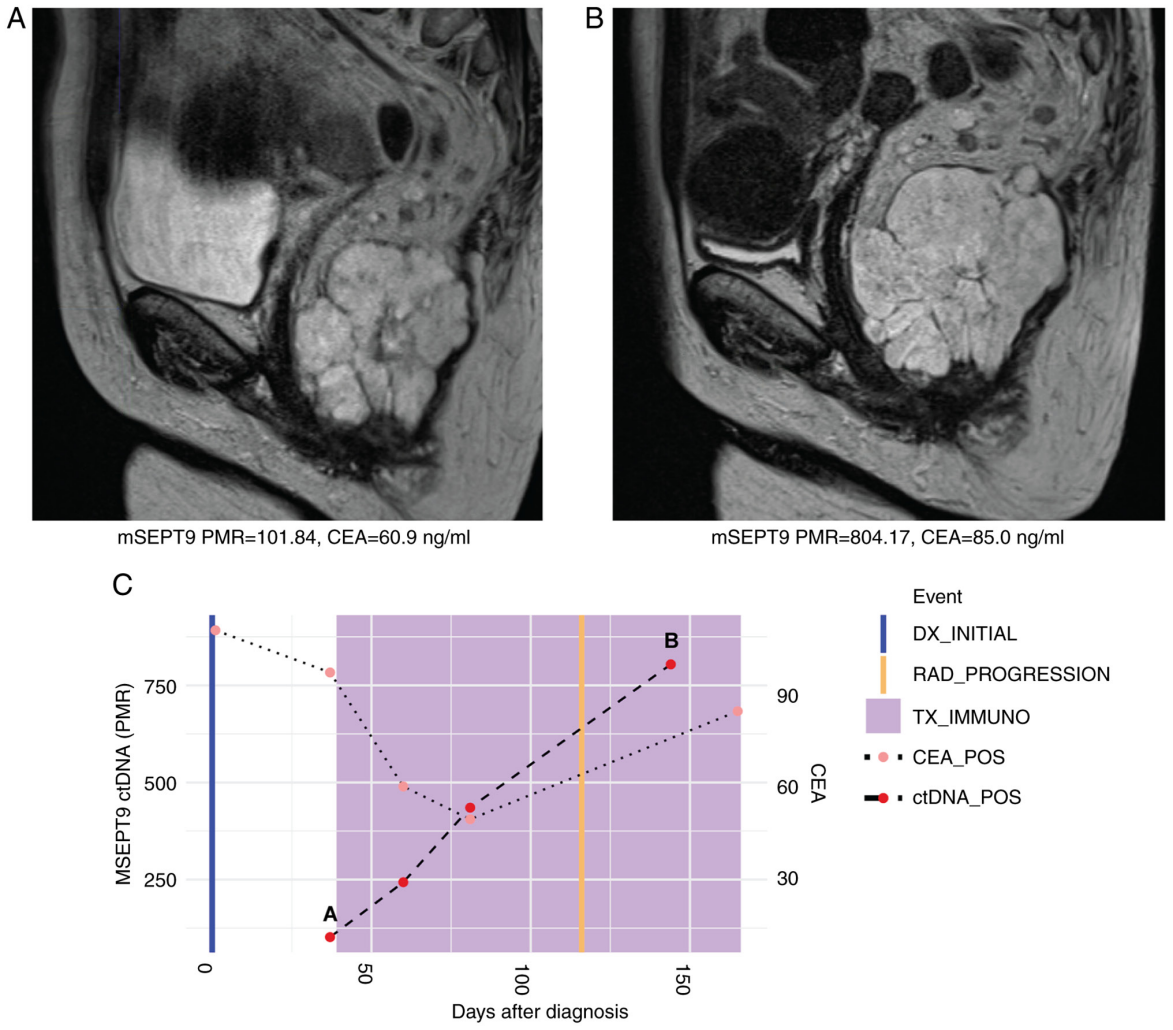
Case 2 with microsatellite instability-high metastatic rectal cancer shows rise in ctDNA levels consistent with radiographic and clinical progression to immunotherapy. (A) MRI at initiation of immunotherapy treatment showing primary rectal tumor. Beneath, mSEPT9 ctDNA and CEA levels from blood drawn at this timepoint. (B) MRI at cycle six of pembrolizumab showed growth in size of primary rectal tumor. Beneath, ctDNA and CEA levels from blood draw at this timepoint increased. (C) Timeline of mSEPT9 and CEA levels over immunotherapy course. The timepoints matching MRI panels A and B above are shown on the timeline graph. Rising mSEPT9 ctDNA and CEA levels between initiation and cycle six of immunotherapy corresponded with radiographic and clinical progression in the primary rectal tumor. mSEPT9, methylated *SEPTIN9* gene; PMR, percentage of methylation reference; CEA, carcinoembryonic antigen; DX, diagnosis; RAD, radiologic; TX_IMMUNO, treatment with immunotherapy; POS, positive; ctDNA, circulating tumor DNA; MRI, magnetic resonance imaging.

A 60-year-old woman with MSI-H metastatic rectal cancer to the lungs was initiated on first-line pembrolizumab (200 mg every 3 weeks). Plasma mSEPT9 ctDNA was positive at treatment initiation with a PMR of 101.84. The PMR increased to 804.17 by cycle 6 of pembrolizumab, which corresponded to an increase in size of the primary rectal tumor on magnetic resonance imaging (MRI). On clinical assessment, there were findings consistent with clinical progression of disease as well. Levels of CEA increased from 60.9 to 85.0 ng/ml during these same timepoints corroborating disease progression to pembrolizumab therapy (Fig. 2). Levels of ctDNA and CEA continued to rise post-radiographic progression (Fig. 2C).

### Case 3

**Figure 3:**
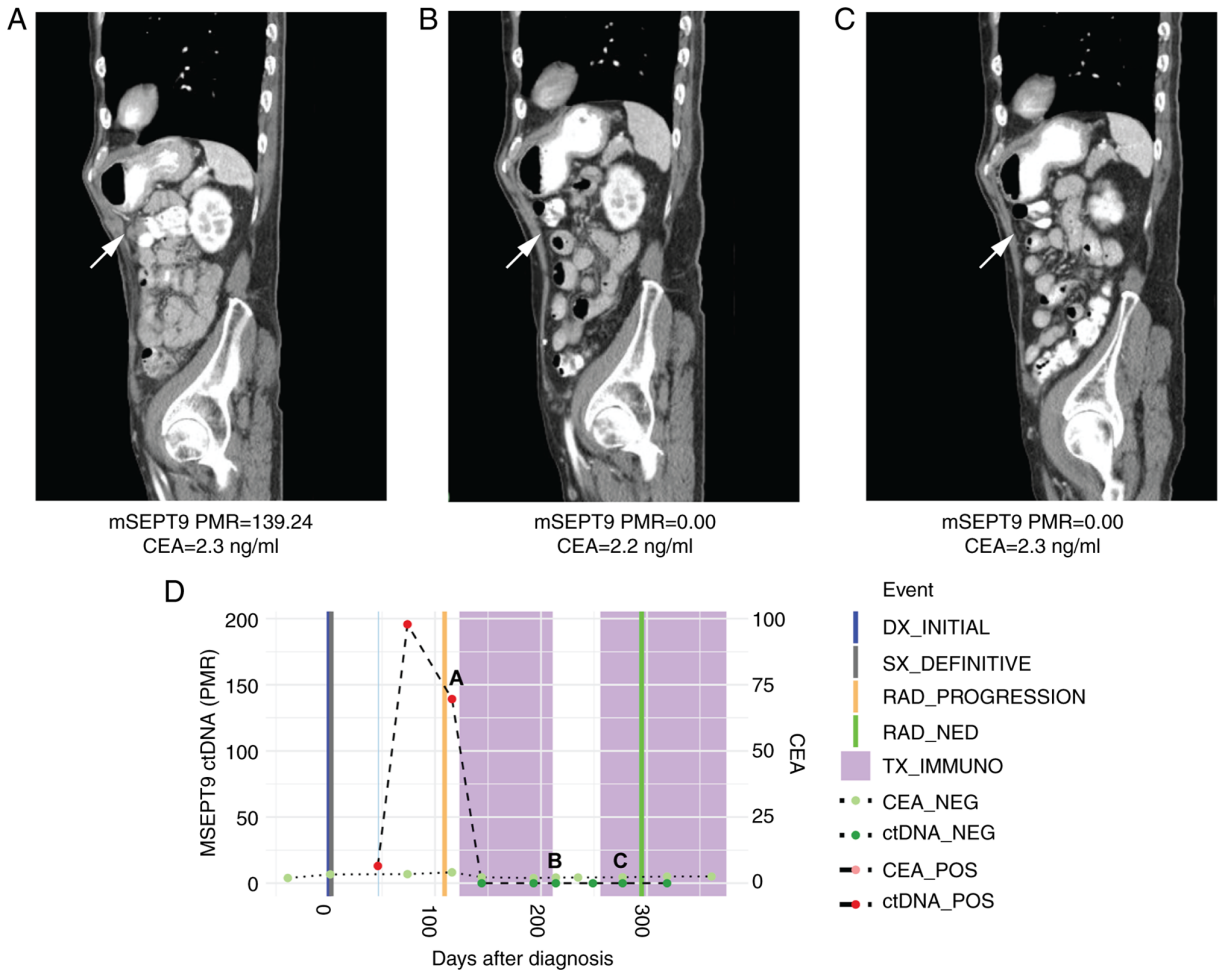
Case 3 with microsatellite instability-high metastatic colorectal cancer shows decline in ctDNA levels consistent with response to immunotherapy in a non-CEA producer. (A) CT scan at initiation of immunotherapy treatment showing peritoneal metastases. Beneath, mSEPT9 ctDNA and CEA levels from blood drawn at this timepoint. (B) CT scan by cycle four of pembrolizumab showed a complete radiographic response in peritoneal metastases. Beneath, ctDNA levels normalized, while CEA levels remained low. (C) CT scan by cycle seven of pembrolizumab showed a sustained complete radiographic response in peritoneal metastases. Beneath, ctDNA levels remained normalized, while CEA levels remained low. (D) Timeline of mSEPT9 and CEA levels over immunotherapy course. The timepoints matching CT panels A, B, and C above are shown on the timeline graph. Compared with initiation, mSEPT9 ctDNA levels normalized by cycle four and cycle seven of immunotherapy, which correlated with response to treatment; CEA levels remained low through all three timepoints. mSEPT9, methylated *SEPTIN9* gene; PMR, percentage of methylation reference; CEA, carcinoembryonic antigen; DX, diagnosis; SX, surgery; RAD, radiologic; NED, no evidence of disease; TX_IMMUNO, treatment with immunotherapy; NEG, negative; POS, positive; ctDNA, circulating tumor DNA; CT, computed tomography.

A 74-year-old man with MSI-H colon cancer metastatic to the abdominal wall and peritoneum who progressed on adjuvant 5-fluorouracil and oxaliplatin (FOLFOX) was treated with pembrolizumab (200 mg every 3 weeks). He was plasma mSEPT9 ctDNA positive at cycle 1 of immunotherapy (PMR 139.24). However, by cycle 4 of pembrolizumab, he became ctDNA negative and remained negative by cycle 7 (PMR 0 for both timepoints), which corresponded to a complete radiographic response at these same timepoints (Fig. 3). Notably, serial CEAs throughout his pembrolizumab treatment remained low at 2.2-2.3 ng/ml.

### Case 4

**Figure 4:**
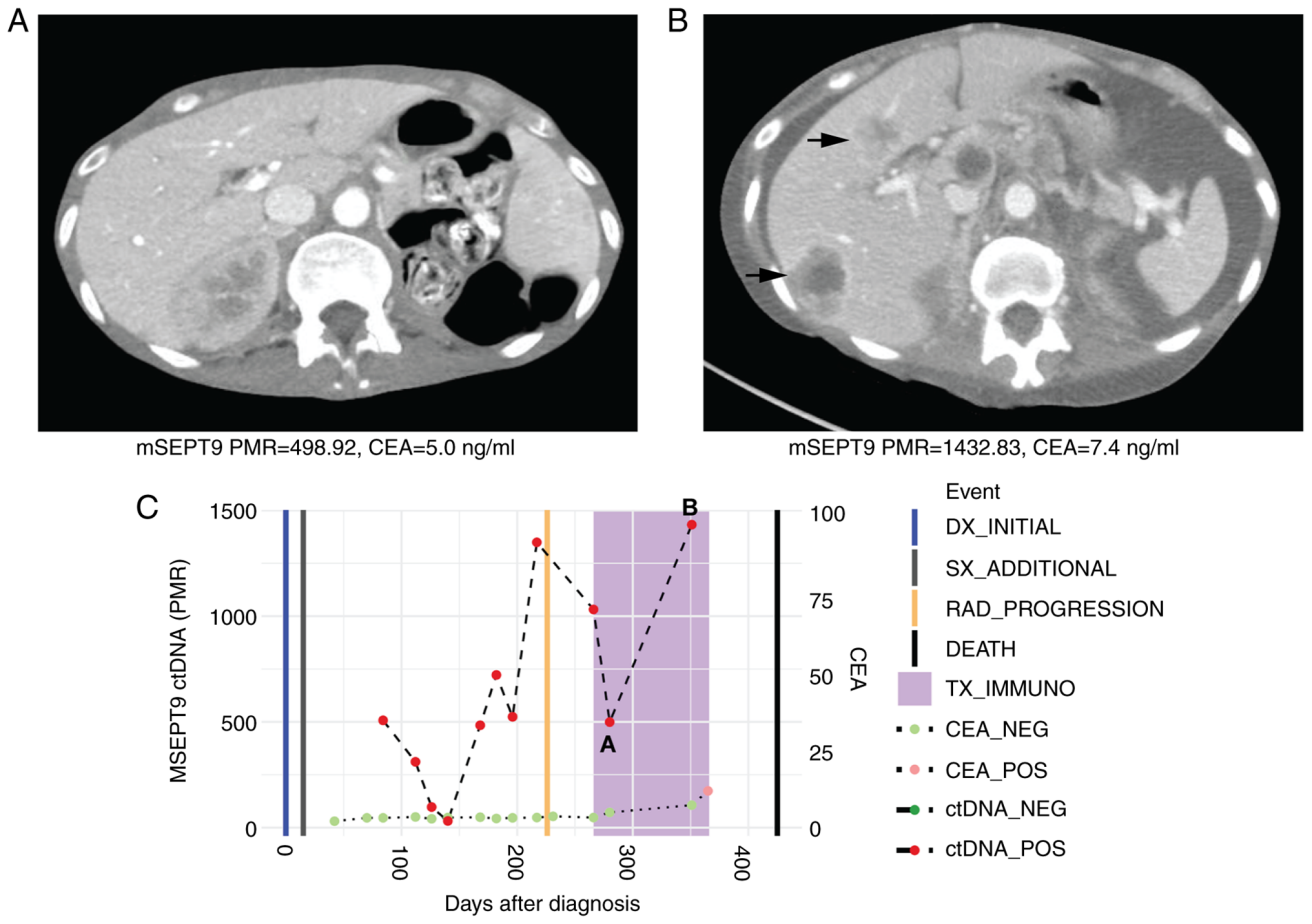
Case 4 with microsatellite stable metastatic colorectal cancer shows rise in ctDNA levels consistent with radiographic progression to immunotherapy. (A) CT scan at initiation of immunotherapy treatment showed normal liver. Beneath, mSEPT9 ctDNA and CEA levels from blood drawn at this timepoint. (B) CT scan after four cycles of third-line regorafenib and nivolumab showed radiographic progression with development of liver metastases. Beneath, ctDNA and CEA levels from blood drawn at this timepoint increased. (C) Timeline of mSEPT9 and CEA levels over immunotherapy course. The timepoints matching CT panels A and B above are shown on the timeline graph. Rising mSEPT9 ctDNA and CEA levels between initiation and post-cycle four of immunotherapy correlated with disease progression. mSEPT9, methylated *SEPTIN9* gene; PMR, percentage of methylation reference; CEA, carcinoembryonic antigen; DX, diagnosis; SX, surgery; RAD, radiologic; TX_IMMUNO, treatment with immunotherapy; NEG, negative; POS, positive; ctDNA, circulating tumor DNA; CT, computed tomography.

A 55-year-old woman with treatment-refractory, unresectable colon cancer that was MSS was treated with third-line regorafenib (80 mg oral once a day for 21 days every 28-day cycles) and nivolumab (240 mg intravenously every 2 weeks). Following 4 cycles of regorafenib and nivolumab, her plasma mSEPT9 ctDNA values (PMR) increased from 498.92 to 1432.83 with marked disease progression on interval CT scans as evidenced by new hepatic metastases (Fig. 4). She died 2 months later due to progressive disease. Her CEA levels were uninformative and ranged from 5.0-7.4 ng/ml during her immunotherapy course.

## Discussion

To the best of our knowledge, there have been only 3 studies to demonstrate that rises and declines in plasma ctDNA levels predicted radiographic tumor progression and response, respectively, in MSS or MSI-H mCRC treated with immunotherapy (9-11). In one study, changes in plasma ctDNA levels 4 weeks from initiation of immunotherapy was predictive of radiographic response, while undetectable ctDNA at week 8 from immunotherapy was associated with prolonged progression-free survival and overall survival in another study (9,11).

Our case series supports the use of plasma ctDNA as a dynamic marker of response to immunotherapy in mCRC. In all cases, a decrease or clearance of ctDNA corroborated radiographic tumor response, while rises in plasma ctDNA levels corroborated radiographic tumor progression to immunotherapy. In cases where colorectal tumors produced CEA, ctDNA levels aligned with changes in CEA, and furthermore, both were in concordance with radiographic response assessments. Our findings are consistent with the literature showing that plasma ctDNA correlates with conventional measures of tumor response to systemic therapy (by CEA and imaging) in mCRC (12). However, unlike CEA, wherein measurable levels can be found in normal individuals but a proportion of colorectal tumors can be non-secretors of CEA, ctDNA as a measure of minimal residual disease (MRD) is a binary metric whose presence indicates that a large burden of residual metastatic cancer cells remain in one's body (13). It is therefore not surprising that there is growing evidence to suggest that ctDNA demonstrates superior sensitivity to CEA to detect recurrence in non-metastatic CRC and radiographic progression events in mCRC (13-16). In mCRC, changes in ctDNA levels from pretreatment to cycle 2 was a better predictor of radiologic response to chemotherapy than CEA at this early time point (17). In resected, stage I-III CRC, ctDNA detection offers median lead times of up to 11 months for radiographic detection of recurrence (18). A recent large observational cohort demonstrated that in those with postoperative ctDNA positivity following curative-intent surgery for stage I-IV CRC, clearance of MRD with adjuvant chemotherapy was also a positive prognostic indicator (19).

Importantly, we report a novel finding that plasma ctDNA levels predicted radiographic response (or lack of) to immunotherapy in CEA non-producers. CEA represents the only conventional and recognized blood-based test for monitoring response to systemic therapy in mCRC. Notably, up to 34% of patients with CRC are CEA non-producers, which represents a clinically relevant proportion of patients without a marker to monitor systemic treatment response short of interval surveillance imaging (6). In Case 3, plasma mSEPT9 ctDNA levels were detectable at baseline but subsequently cleared over the course of treatment with pembrolizumab and corresponded to a complete radiographic response by cycle 4 of immunotherapy (Fig. 3). CEA was uninformative in this case as the patient had low values of CEA throughout the course of immunotherapy (non-producer). Conversely, in Case 4, CEA levels were fairly low and would not have indicated the degree of rapid disease progression that was detected by interval imaging and a substantial rise in plasma ctDNA levels from baseline to cycle 4 of immunotherapy. This patient died from progressive disease shortly thereafter.

When purposed for detection of tumor mutations through targeted next-generation sequencing (NGS), ctDNA can be used to track dynamic changes in mutations in response to systemic therapy to detect presence of resistance mutations (12). However, in our series we have used a tumor-agnostic, methylated ctDNA marker (mSEPT9) and therefore its assessment of systemic tumor burden is not influenced by tumor mutational profile or prior therapies. When purposed for MRD detection, the timing of therapy to the collection of blood for ctDNA measurement can influence ctDNA levels. For example, surgery can lead to elevation in ctDNA levels lasting as long as 4 weeks from surgery, which is why many groups have recommended postoperative measures of ctDNA to not occur until 4 weeks after CRC surgery (12,20). Chemotherapy can also cause changes in ctDNA as early as 48 h of chemotherapy infusion, although in another series most changes in ctDNA occurred by cycle 2 of chemotherapy (17,21). Early spikes in ctDNA levels within the first few days of chemotherapy infusion may represent a rapid release of ctDNA from lysing tumors (17). The collection of blood samples in our 4 cases for ctDNA assessments occurred prior to the infusion of immunotherapy and before the first dose of oral chemotherapy on each day 1 of the respective cycle of therapy.

Although promising and durable responses have been observed with immune checkpoint blockade in MSI-H mCRC, it should be noted that the median time to onset of response is 2.2 months (range 1.8 to 18.8 months) (1). Therefore, during this initial critical period up until the first interval assessment scan, having a convenient and minimally invasive biomarker that can reliably monitor tumor response to immunotherapy can be of immense benefit to the physician while easing patient anxiety, particularly in the absence of high CEA levels to begin with. This holds especially true in non-metastatic settings where neoadjuvant immunotherapy has been used in MSI-H locally advanced rectal cancer given increasing evidence that these tumors are fairly resistant to standard chemotherapy and chemoradiation (22). Neoadjuvant immunotherapy has been explored in early-stage colon cancers as well (23). Here, serial plasma ctDNA assessments might provide an early indicator of benefit to neoadjuvant immunotherapy and potentially allow a timely transition to salvage chemoradiation or surgery, while preserving a curative intent treatment pathway in the event of lack of tumor response to immunotherapy.

Our group of mCRC cases demonstrates that longitudinal plasma ctDNA assessments can predict true progressors to immunotherapy, even in the context of a CEA non-producing tumor. This is noteworthy, given that there are increasing efforts to consistently detect the phenomenon of pseudoprogression to immunotherapy (24). As evidence accumulates in support of ctDNA being a more sensitive measure of changes in tumor burden than conventional approaches in CRC, it would be prudent to further investigate its role in the detection of pseudoprogression and true progression in larger, prospective cohorts of mCRC patients treated with immunotherapy (7). There are ongoing prospective studies seeking to evaluate the impact of tumor response assessments using plasma ctDNA assays in patients with mCRC and other advanced solid tumors treated with immunotherapy that will hopefully provide more insight in this context (NCT04761783).

Plasma ctDNA represents a minimally invasive tool with promising potential as a measure of tumor burden and tumor response to systemic therapies in CRC. In this case series, we demonstrate that blood-based assessments of mSEPT9 ctDNA predicted radiographic response to immunotherapy in patients with mCRC that were MSS or MSI-H. Interestingly, plasma ctDNA was a predictor of response to immunotherapy even in colorectal tumors that were CEA non-producers. Our findings add to ongoing efforts to establish the role of plasma ctDNA in monitoring response to immunotherapy in CRC. Future studies are warranted to investigate the potential of plasma ctDNA to detect hyperprogressors or differentiate true progression from pseudoprogression in the context of immunotherapy-based treatments in CRC.

## Data Availability

All data generated or analyzed during this study are included in this published article.
